# Evaluation of TRNG Bit Distribution via Stable Entropy Source Synchronization on FPGA

**DOI:** 10.3390/e28010031

**Published:** 2025-12-26

**Authors:** Ryoichi Sato, Mitsuki Fujiwara, Yasuyuki Nogami, Md Arshad Ali, Yuta Kodera

**Affiliations:** 1 Graduate School of Environmental, Life, Natural Science and Technology, Okayama University, Okayama 700-8530, Japan; mitsuki_fujiwara@s.okayama-u.ac.jp (M.F.); yasuyuki.nogami@okayama-u.ac.jp (Y.N.); yuta_kodera@okayama-u.ac.jp (Y.K.); 2 Faculty of CSE, Hajee Mohammad Danesh Science and Technology University, Dinajpur 5200, Bangladesh; arshad@hstu.ac.bd

**Keywords:** TRNG, ring oscillator, FPGA, synchronous circuit, bit distribution, metastable state, multiple D-FFs

## Abstract

This study examined the correlation between the number of delay flip-flops (D-FFs) connected after each ring oscillator (RO) and the bit distribution of random number sequences in an RO-based random number generator (RNG). In our previous research, unstable input signals to the XOR gate contributed to differences in bit distribution. Based on these results, we simulated how combining signals with biased distributions through XOR gates affects the overall bit distribution. Beyond this, we also conducted simulations where the inputs to the XOR gate included not just {0, 1} signals, but also three-state signals incorporating metastable states. We then proposed using multi-D-FFs as synchronization circuits for RO signals and performed analyses on RO-based RNG implementations by estimating metastable output conditions and conducting NIST Special Publication 800-22 tests regarding bit distributions. These results confirm that inserting two or more D-FFs after RO signals improves the bit distribution of RO-based RNG implementations.

## 1. Introduction

The Internet of Things (IoT) has fully transitioned from the research phase to practical implementation, finding widespread adoption across diverse industries, including manufacturing, logistics, and energy management. Advancements and cost reductions in single-board computers and microcontrollers have driven this progress, while FPGAs—through semiconductor scaling and architectural innovation—have achieved over 10,000-fold increases in logic capacity along with more than 1000-fold improvements in both cost reduction and power efficiency [1].

This transformation has enabled devices that were previously used exclusively for research and prototyping to become practical solutions for edge AI and real-time video processing applications. Notably, in recent years, there have been increasingly frequent reports of security incidents, making it an urgent priority to address cybersecurity measures. In securing systems, we must evaluate them based on the three fundamental principles of information security: confidentiality, integrity, and availability. In industrial applications, the protection of confidentiality and integrity becomes essential when continuously acquiring video and image data. Consequently, research is underway to implement hardware optimizations for cryptographic algorithms such as ChaCha20-Poly1305 stream encryption and the AES algorithm on FPGAs [2,3]. These methods are crucial for encrypting communication channels, while research also exists on content-based security—such as images and videos. Such information may unintentionally contain sensitive privacy data, and research is progressing to develop encryption technologies to protect this information [4,5]. Beyond ensuring the security of communication pathways, we also need to protect the information flowing through them, making comprehensive approaches across multiple dimensions necessary to comply with zero-trust principles.

However, IoT devices face significantly more resource constraints than traditional computers, and numerous security vulnerabilities have been reported [6]. In recent years, physical attack methods such as side-channel attacks (SCAs) have also been documented [7]. Therefore, FPGAs—which are indispensable for efficiency gains, power reduction, and performance enhancement—and the design of cryptographic circuits along with the quality of random numbers used for these purposes will become critical considerations in securing IoT systems of the future.

Contemporary cryptographic systems require cryptographically secure pseudo-random number generators (CSPRNGs) that demonstrate both statistical randomness and computational unpredictability [8]. However, the security of CSPRNGs fundamentally depends on the quality of their seed values, because poor-quality seeds can compromise the entire cryptographic system regardless of the algorithmic sophistication of the CSPRNG itself. This dependency demands a critical requirement for high-quality sources of entropy. Consequently, the seed requires information-theoretic security to maintain its security guarantees of CSPRNGs [9]. To meet information-theoretic security, true random number generators (TRNGs) are commonly used, and the quality of the random numbers is affected by handling the unstable phenomena that are used as their analog noise sources.

We focus on TRNGs that utilize ring oscillators (ROs) as analog noise sources. ROs can be efficiently implemented in FPGAs, where their oscillation frequencies exhibit inherent jitter arising from thermal noise and semiconductor variability [10,11,12]. This jitter serves as a noise source for random number generation. Major processor manufacturers have validated this approach: ARM and AMD both deploy RO-based entropy sources with various oscillator lengths [13,14], and both implementations comply with the NIST SP 800-90B standards [15]. These commercial implementations demonstrate the practical significance and viability of RO-based RNGs for production security systems. Given the critical role of entropy sources in cryptographic security, RO-based RNGs must maintain a consistently high-entropy output to provide robust seeds for cryptographic applications. This study focuses on TRNGs as the foundational component of secure random number generation, with the primary objective of enabling these systems to produce stable, high-quality entropy values suitable for resource-constrained IoT environments.

RO-based RNGs can function as TRNGs by combining ROs with XOR operations. Although adding more RO circuits improves randomness in this design, there is a challenge in that no significant improvement in randomness is observed when using more than 10 RO circuits [16]. In this study, we focused on the fact that RO circuits are asynchronous with the onboard clock on FPGAs and hypothesized that metastable states might exist in the input signals to lookup tables (LUTs). Wold et al. [11] reported that inserting delay flip-flops (D-FFs) after ROs improved bit distribution. However, they did not address the metastable states. Therefore, we investigated the impact of metastable states on XOR operation. Fujiwara et al. [17] identified an unusual bias in the XOR gate output bit distribution depending on whether a single D-FF was inserted between the RO and the XOR gate and attributed this to the metastable state phenomenon. Based on these findings, we hypothesized that reducing the number of signals in metastable states at the XOR gate would maximize the effectiveness of noise source coupling within the XOR gate. In this study, we implemented an RO-based RNG using multiple D-FFs (multi-D-FFs) to verify whether bit distribution improves as an entropy source for RO-based RNG implementation. Through simulations and experiments, we confirmed that inserting two or more D-FFs between the RO and the XOR gate results in improved bit distribution.

Section 2 presents fundamental background knowledge, while Section 3 describes our previous research and simulation results based on bit emergence probabilities. In Section 4, we verify the bit distribution when multi-D-FFs are applied using the same experimental setup as in [17]. In Section 5, we set the number of multi-D-FFs stages to {0, 1, 2, 3, 4} and constructed small-scale random number generation circuits.

## 2. Fundamentals

This study focuses on an RO-based RNG, which is a physical random number generator that can be implemented on an FPGA. Field-Programmable Gate Arrays (FPGAs) are chips capable of building logical circuits, and in development, ensuring proper calculations through synchronization techniques and avoiding unstable circuit implementations becomes crucial. Conversely, there are also approaches where creating such structures intentionally generates instability and is utilized for applications like random number generation. This section briefly explains the principles of the ring oscillator-based random number generator (RO-based RNG) used in this research and describes the metastability issues arising when synchronizing asynchronous circuits.

### 2.1. RO-Based RNG

**Figure 1:**
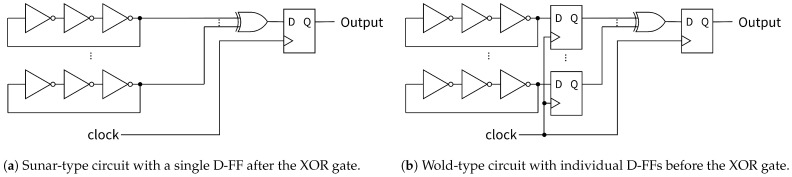
RO-based RNG circuit architectures.

Sunar et al. [10] proposed a methodology for constructing random number generation circuits based on ROs (Figure 1a). In their method, multiple ROs are directly connected to an XOR gate, and the XOR output is then synchronized with a single D-FF using a clock to generate random number sequences. Furthermore, Wold et al. [11] proposed an improved TRNG design by inserting a D-FF between each RO and the XOR gate (Figure 1b). In the Wold-type circuit, each RO is first synchronized individually by one D-FF before being combined with the XOR gate, and the synchronized XOR output is used as a random number sequence. The key difference between these architectures is the synchronization timing: Sunar’s method synchronizes the combined signal after the XOR operation, while Wold’s method synchronizes each RO signal individually before the XOR operation. In both architectures, the bit rate depends on the clock frequency used for synchronization.

An RO is an oscillation circuit, and its frequency is unstable because it includes jitter. It is constructed using odd NOT gates connected in a ring shape. The oscillation frequency is affected by the wiring length and the number of NOT gates. Let $v_{wire}$ denote the propagation speed in the FPGA wiring and $t_{NOT}$ denote the time taken for the signal level to switch at each NOT gate. The propagation delay in an RO wiring $t_{wire}$ is calculated using the wire lengths $l_{wire}$ and $v_{wire}$ as follows. Then, when designing an RO consisting of NOT gates $n_{NOT}$, the oscillation frequency $f_{RO\text{-}ideal}$ is calculated by

(1) $$f_{RO\text{-}ideal}=\frac{1}{t_{wire}+n_{NOT}t_{NOT}}=\frac{1}{\frac{l_{RO}}{v_{wire}}+n_{NOT}t_{NOT}}.$$

Equation (1) is an ideal environment that does not include external noise and manufacturing errors. Each semiconductor in an FPGA has individual differences. Therefore, the respective NOT gates in an RO have different switching delays and temperature characteristics. According to [18], $t_{NOT}$ is listed as a combinatorial delay, and only the maximum delay time is specified. Therefore, let α and $t_{NOT\text{-}i}$ be the errors due to the semiconductor characteristics and a certain NOT gate switching delay, respectively. The switching delay $t_{NOT\text{-}i}$ is as follows:

(2) $$t_{NOT\text{-}i}=t_{NOT}+\alpha_{i}.$$

Let β be a delay due to external errors, which includes external power noise and environments. The reality frequency $f_{RO}$ is based on (1) as follows:

(3) $$f_{RO}=\frac{1}{\frac{l_{RO}}{v_{wire}}+\sum_{i=1}^{n_{NOT}}\left(t_{NOT}+\alpha_{i}\right)+\beta }.$$

The variables α and β are not predictable and dynamic; therefore, the oscillation frequency is always unstable. The frequency deviation is called jitter and is used as a noise source in a TRNG.

An RO-based RNG collects and combines multiple RO outputs via an XOR gate. An RO is synchronized by an FPGA onboard clock before the signal is inputted to the XOR gate. If each RO is not synchronized by an FPGA onboard clock, the random number sequence of the RO-based RNG exhibits a bit distribution deviation [11,17]. The output of the XOR gate is used as a random number sequence.

This study focuses on an asynchronous RO with an FPGA onboard clock. The next section explains the instability phenomenon in logic gate construction, which is different from jitter.

### 2.2. Metastability

Metastability refers to the phenomenon where a system probabilistically converges to either a High or Low state from a metastable state, which is characterized by an intermediate voltage level that is neither High nor Low.

**Figure 2:**
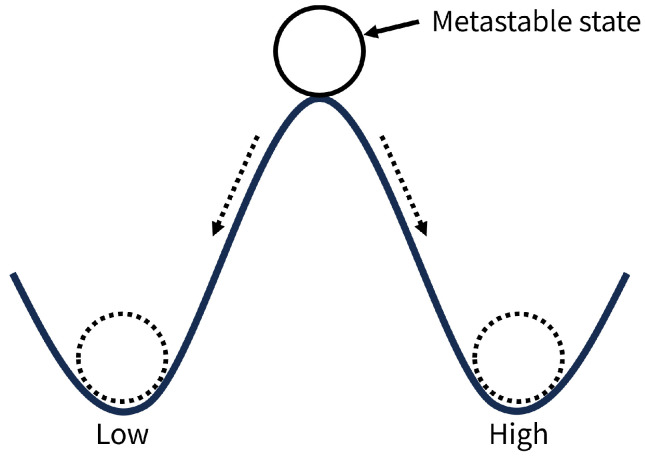
Idea of metastable state using ball and hill.

The metastable state is often explained using the analogy of a ball and hill [19,20]. In Figure 2, a ball balanced at the top of a hill corresponds to a metastable state. The ball eventually rolls down either the left or right slope to determine the final value; however, both the time required for convergence and the direction of convergence are probabilistic. This probabilistic convergence phenomenon is called metastability.

A representative example of this phenomenon is signal transfer between asynchronous clock domains. Because the signals have different frequencies and phases, the timing of the signal capture by D-FFs can sometimes result in a metastable state. Although the occurrence of this phenomenon itself is probabilistic, once a metastable state is entered, it can potentially cause adverse effects on downstream circuit operations. To mitigate this issue, it is recommended to use synchronization circuits consisting of at least two or more D-FFs when synchronizing asynchronous signals [19].

In general circuit design, avoiding this phenomenon is crucial for ensuring system reliability. However, in physical random number generation, this phenomenon can be exploited as a noise source. Hata et al. [21] presented a method for generating physical random numbers using a phenomenon in SR latches. This approach waits for the value to converge to either High or Low after metastability occurs and then uses the converged value as a random number. Another approach that exploits metastability is the transition effect ring oscillator (TERO), which generates random bits by detecting variations in oscillation transient times [22]. Similarly, in the RO-based RNG employed in this study, metastability occurs when synchronizing the unstable periods of the ROs with the FPGA onboard clock. Managing this phenomenon has a substantial impact on research outcomes.

## 3. Analysis and Hypotheses Regarding Bit Appearance Probability

In this session, we describe our previous studies, the XOR gate in the RO-based random number generator and unstable signal inputs to the LUT. Based on these works, the bit occurrence probability of the XOR gate was simulated with and without metastable states.

### 3.1. The Effect of XOR Gate on Bit Distribution

The RO-based RNG output is a collection of each RO analog noise source using an XOR gate. Here, we focus on why the method for collecting analog noise sources uses the XOR gate. To achieve this, we compared the periodicity by synthesizing one RO and two ROs using an XOR gate [23]. To verify this, we analyzed the output values from the RO-based RNG using two methods: visual confirmation through graph plots of random sequences and analysis of state transition probabilities using scatter plots based on the Markov process. Both methods were designed to examine the periodicity. By analyzing the periodicity of the output values, we can determine how the presence or absence of an XOR gate affects periodicity. Visual confirmation revealed that synthesizing the two ROs with an XOR gate resulted in more complex bit patterns. Using Markov process verification, we attempted to investigate whether combining two ROs via an XOR gate reduces the skew in state transition probabilities. Both verifications indicate that the periodicity becomes more difficult to discern. Therefore, combining two ROs via an XOR gate results in a more complex period than that of a single RO.

Furthermore, this study discusses the reason for the increased complexity [23]. An RO is an oscillation circuit that generates a signal that oscillates at a frequency given by Equation (3). The signal synthesized by combining each RO via the XOR gate resulted in superposition of the oscillating signals. This study states that the frequency of the synthesized signal becomes the frequency of the least common multiple of each frequency. The frequency of RO is significantly influenced by the wiring length and number of NOT gates. For the entropy of an RO-based RNG, using ROs with similar frequency values contributes to the complexity of the frequency of the synthesized signal. Therefore, the use of ROs with identical placements and wiring is recommended.

### 3.2. Relationship Between an Unstable Value and LUT

In [11], it was shown that connecting a D-FF after each RO causes an even bit distribution in the output random numbers. According to the authors’ analysis of this phenomenon, it was reported that the signals became synchronized with the sampling clock, resolving setup, and hold time. They validated this by demonstrating the equalization of the bit distribution. However, if D-FFs are used to synchronize asynchronous signals, the potential occurrence of metastable states is not considered.

In [17], our previous study hypothesized that whether the input signal is synchronized with a stable clock or not affects the XOR operation. Therefore, we conducted experiments focusing on a single D-FF, a synchronization circuit, for this investigation. Additionally, the relationship between the D-FF and bit distribution in the RO-based RNG sequence is discussed. We refer to the necessity of D-FF in terms of the discrepancy in bit distribution when the intermediate potentional signal is input to the LUT. One side of the XOR gate connects to a constant value of ‘0’ or ‘1’, whereas the other side connects to an RO with or without a D-FF. When a D-FF is not applied to the RO, there is a percentage point difference in the output probabilities between the constant ‘0’ case and the constant ‘1’ case. However, when a D-FF is applied to RO, this percentage point difference is reduced compared to the case without a D-FF. This result shows that a stable input to the LUT influences the uniform bit distribution. Therefore, employing a D-FF between the RO and XOR gate in the RO-based RNG on an FPGA leads to a uniform bit distribution by protecting the input intermediate potentional signal in the XOR gate.

### 3.3. XOR Output and Bit Appearance Probability

We considered the effect of the bit distribution from the entropy source on the bit distribution of the XOR gate output in Section 3.1. There are two primary factors contributing to this phenomenon: First, when threshold voltages $V_{th}$ of individual components do not lie exactly between the high and low voltage levels. Second, when the metastable state occurrence introduces biasing in the LUT outputs. For the random number sequence test, statistical randomness tests, including NIST Special Publication 800-22 (NIST SP 800-22), assume a uniform bit distribution. Therefore, achieving a uniform bit distribution is essential for RO-based RNGs. RO-based RNGs generate random numbers based on oscillation signals that include jitter, making it difficult to predict the signals input to the XOR gate. Based on this, this study considers the input signals to the XOR gate as probabilistic events. At this point, we simulate the output bit distribution of the XOR gate using a binomial distribution and confirm the bit distribution of the RO output, the number of ROs, and their relationship.

This validation approach treats the input signals to the XOR operation as stochastic processes. Let the sequence generated from an analog noise source be considered a stochastic process. As an example, let $x_{i}\in \left\{0,1\right\}$ represent a particular bit, we denote a random sequence of length 2 as $X_{2}=x_{1}x_{0}$. Here, it is assumed that $x_{1}$ is generated after $x_{0}$. When $x_{1}$ is dependent on $x_{0}$, the probability of observing this random sequence $X_{2}$ is given by

(4) $$P\left(X_{2}\right)=P\left(x_{1},x_{0}\right)=P\left(x_{1}|x_{0}\right)P\left(x_{0}\right).$$

If instead $x_{0}$ and $x_{1}$ are independent, this expression can be rewritten as

(5) $$P\left(X_{2}\right)=P\left(x_{1}\right)P\left(x_{0}\right).$$

The defining characteristics of true random numbers are non-periodicity and unpredictability. Regarding the bit length l∈N, each bit $x_{i},i\in 0,\cdots ,l-1$ of the true random sequence $X_{l}$ can be treated as independent. The probability of occurrence for the true random sequence $X_{l}$ then becomes

(6) $$P\left(X_{l}\right)=\prod_{i=0}^{l-1}P\left(x_{i}\right)=\prod^{l}P\left(x\right).$$

Estimating the probability of each bit appearance in this analog noise source proves challenging. Since each bit’s probability is independent, we can interpret the values obtained during digitization as probabilistically sampled from the population. By collecting l bit samples from the population and estimating the population characteristics, we can derive the probability of occurrence for each bit. The probability of observing 1 can be calculated based on the Hamming weight $W(X_{l})$ of the *l*-bit random sequence. Therefore, the bit occurrence probability is estimated as follows:

(7) $$W\left(X_{l}\right)=\sum_{i=0}^{l-1}x_{i}.$$

Fujiwara et al. [17] calculate the probability of 0 occurrences, but using Equations (6) and (7), the probabilities of individual bit appearances can be interpreted as those generated by the RO output. Accordingly, if we conceptualize the analog noise source as an output of an RO circuit, the following holds true:

(8) $$p_{RO}=\frac{W(X_{l})}{n(X_{l})}=\frac{1}{l}\sum_{i=0}^{l-1}x_{i}.$$

Equation (8) shows the probability of inputting a signal to the XOR gate. In the RO-based RNG, multiple ROs are connected with an XOR gate. Here, we assume that each RO input to the XOR has the same probability. Let N∈N be the number of ROs and let j∈1,⋯,N be the iterator for the *j*-th RO. Furthermore, let $p_{RO\text{-}j}\in \left[0,1\right]$ be the probability of 1 appearing from the *j*-th RO. Let k∈N be the input value for an XOR operation. Then, the probability that *k* inputs out of *N* ROs is 1 as

(9) $$P\left(x=k\right)=\left(\begin{array}{c} N\\ k \end{array}\right){p_{RO\text{-}j}}^{k}{\left(1-p_{RO\text{-}j}\right)}^{N-k}$$

using a binomial distribution. The probability of 1 from each RO is considered as an independent event. We assume that the appearance probability of 1 from all ROs is the same as follows:

(10) $$p_{RO}=p_{RO\text{-}1}=\cdots =p_{RO\text{-}n}.$$

Denoting $p_{XOR}$ as the probability of 1 appearing in the result of the XOR operation, from Equations (9) and (10) it follows that

(11) $$p_{XOR}=\sum_{1\leq j\leq N,j\;\mathrm{is}\;\mathrm{odd}}\left(\begin{array}{c} N\\ j \end{array}\right){p_{RO}}^{j}{\left(1-p_{RO}\right)}^{N-j}.$$

For the simulation, probabilities of 0.642 and 0.587 were used as the probabilities of 1 appearing from RO, as referenced in [17]. In addition, an ideal probability of 0.500 was incorporated into the simulation. We simulated $p_{XOR}$ with these three probabilities as $p_{RO}$ based on Equation (11). The transition of $p_{XOR}$ when *n* was set from 1 to 10 is displayed in the graph. The simulation environment is shown in Table 1.

**Figure 3:**
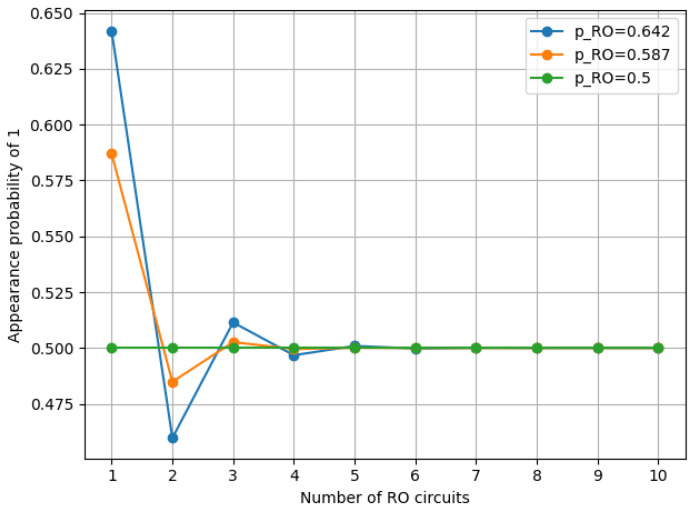
Appearance probability of 1 for XOR output.

Figure 3 shows the simulation results. As shown in the figure, increasing the number of ROs resulted in the convergence of the probability $p_{XOR}$ to 0.500. Furthermore, as the probability $p_{RO}$ approaches the ideal value of 0.500, the probability $p_{XOR}$ converges to 0.5 with a smaller number of ROs.

This simulation showed that the bit distribution of the XOR output is expected to become more uniform as the RO output approaches 0.5. Connecting multi-D-FFs to the RO output prevents it from entering the LUT in a metastable state and improves the bit distribution, as discussed in Section 3.2. Based on these results, we can hypothesize that applying multi-D-FFs to the RO-based RNG affects the bit distribution uniformity of the output random number sequence.

### 3.4. XOR Output and Metastable State

Previously, we performed a probabilistic simulation of the XOR output when the signals input to the XOR gate were {0, 1}. Here, we conduct a simulation that accounts for signals in metastable states that may occur when sampling asynchronous signals.

Digital logic typically operates only with {0, 1}, where binary states correspond to high/low voltage levels within the circuit. However, certain voltage values that do not fall within these high/low ranges may occur. We refer to this voltage state as an “intermediate voltage,” which can cause errors in correct computation within the circuit. To prevent such occurrences, it is necessary to implement synchronization circuits or design the circuit architecture with careful attention to timing constraints.

Let $f_{RO}$ represent the frequency of a particular RO, and let $f_{CLK}$ denote the frequency of the clock on the FPGA board. Here, we define the phase of $f_{RO}$ as $\theta_{RO}\in \left[0,2\pi \right)$. Consider sampling $f_{RO}$ using a D-FF with clock frequency $f_{CLK}$. When a clock frequency and an RO signal are coprime, we can say that the output phase when sampling $f_{RO}$ uniformly distributes within [0,2π) [20]. If we model $f_{RO}$ as a sinusoidal waveform, and when the D-FF performs sampling near the points θ=0,π, this corresponds to being sampled during the setup or hold time at frequency $f_{CLK}$. In this case, the output of the D-FF will enter a metastable state. Building upon this, we can now analyze the probability of entering a metastable state.

Here we consider two probabilistic events:

A Probability of sampling a specific RO oscillation cycle within a given 1-second period for RO signals;
B Probability that the sampling occurs near the points θ=0,π within an RO cycle period.

Letting $t_{RO\text{-}sw}$ represent the switching time interval within the RO signal’s period and $T_{RO}$ denote the complete RO signal period, we can express each probability as follows:

(12) $$\begin{array}{cc} P(A) & =\frac{1}{f_{RO}}, \end{array}$$

(13) $$\begin{array}{cc} P(B) & =\frac{t_{RO\text{-}sw}}{T_{RO}}. \end{array}$$

From Equations (12) and (13), the probability $P_{RO}\left(M\right)$ of entering a metastable state when synchronizing an RO signal with a D-FF is thus

(14) $$P_{RO}\left(M\right)=P\left(B|A\right)=\frac{P(A\cap B)}{P(B)}=\frac{t_{RO\text{-}sw}}{T_{RO}}$$

Next, we examine the probabilities $P_{RO}\left(0\right)$ and $P_{RO}\left(1\right)$ of obtaining 0, 1 when synchronizing an RO signal with a D-FF. From Equations (12) and (13), we can derive the following:

(15) $$P_{RO}\left(0\right)+P_{RO}\left(1\right)=P\left(\bar{B}|A\right)=\frac{T_{RO}-t_{RO\text{-}sw}}{T_{RO}}.$$

Thus, when synchronizing an asynchronous RO signal with the clock via D-FFs, metastable states will occur. The probability of this occurrence can be calculated based on the frequency of the RO signal and its switching time.

Consequently, these three signal patterns—including the metastable state—will now be input to the XOR gate, each occurring with their respective probabilities.

Now, let us consider the output when the three possible inputs (including a metastable state) are applied to the XOR gate. Let XORed denote the output of the 2-input XOR gate with respect to inputs RO1 and RO2. As noted in the literature [17], the exact behavior of the XOR gate when receiving a metastable signal is unclear. For this analysis, we assume that the presence of even a single metastable input causes output instability, which we designate as M. The resulting truth table is shown in Table 2.

Based on these considerations, we will simulate the probability of each bit state in the XOR gate according to the binomial distribution. As in Section 3.3, we assume the probabilities of each bit appearing from each RO to be identical. We further assume that both the sequences emerging from each RO and the individual bit appearance probabilities are all statistically independent. The output of the XOR gate in this case can be determined as follows:

(16) $$\begin{array}{cc} P_{2\text{-}XOR}\left(0\right) & =P_{RO}{\left(0\right)}^{2}+P_{RO}{\left(1\right)}^{2}, \end{array}$$

(17) $$\begin{array}{cc} P_{2\text{-}XOR}\left(1\right) & =2P_{RO}\left(0\right)P_{RO}\left(1\right), \end{array}$$

(18) $$\begin{array}{cc} P_{2\text{-}XOR}\left(M\right) & =P_{RO}{\left(M\right)}^{2}+2P_{RO}\left(M\right)\left(P_{RO}\left(0\right)+P_{RO}\left(1\right)\right). \end{array}$$

Using these results, we will now examine the probabilities when employing N ROs. The outputs of each XOR gate can be represented as follows:

(19) PN-XOR(0)=∑0≤i≤N,i is evenNiPRO(0)N−iPRO(1)i,

(20) PN-XOR(1)=∑0≤i≤N,i is oddNiPRO(0)N−iPRO(1)i,

(21) PN-XOR(M)=∑1≤i≤N{NiPRO(M)iPRO(0)+PRO(1)N−i}.

From Equation (21), we can see that the probability of metastable state occurrence is expected to increase as the number of ROs N increases.

**Figure 4:**
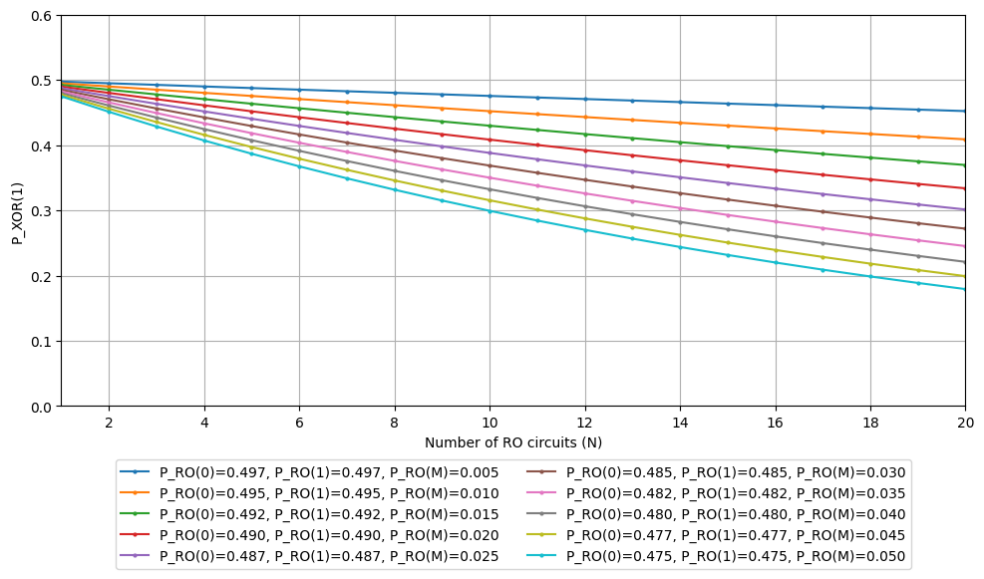
The simulation result of the state 1 appearance probability in the N-XOR gate output.

**Figure 5:**
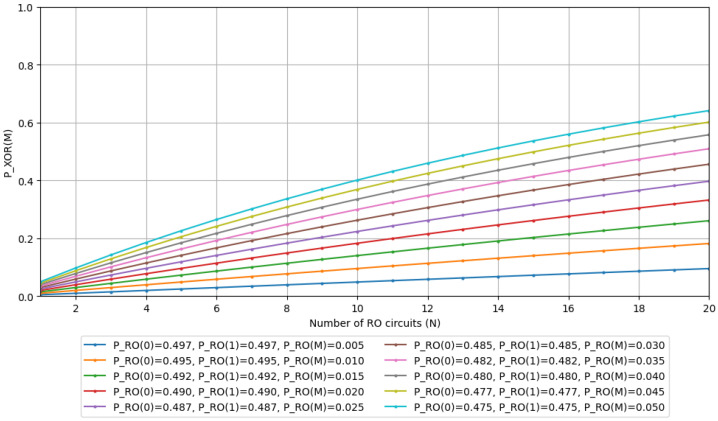
The simulation result of the matastable state appearance probability in the N-input XOR gate output.

We will now conduct simulations based on Equations (19)–(21). The simulation environment is specified in Table 1. For our test parameters, we set $P_{RO}\left(0\right)=P_{RO}\left(1\right)$ and observe the trends when varying $P_{RO}\left(M\right)$. For $P_{RO}\left(M\right)$, let’s assume a switching delay of $t_{RO\text{-}sw}=100\;\mathrm{ps}$, with RO frequencies $f_{RO}=\left\{50\;\mathrm{MHz},100\;\mathrm{MHz},\ldots ,500\;\mathrm{MHz}\right\}$ and clock frequency at 100 MHz. These values serve as reference points for calculating the probability of metastable state occurrences, so we will disregard the specific relationship between $f_{CLK}$ and $f_{RO}$ in this instance. Using the above values and Equation (14), we obtain $P_{RO}\left(M\right)=\left\{0.005,0.010,\ldots ,0.050\right\}$, which we will utilize as simulation parameters. The simulation results for the probabilities of outputs $P_{XOR}\left(1\right)$ and $P_{XOR}\left(M\right)$ are shown in Figure 4 and Figure 5, respectively.

The results clearly show that the probability of metastable state occurrences increases as the number of RO circuits grows. Moreover, when the switching delay $t_{RO\text{-}sw}$ remains constant, the higher the frequency of the RO signals, the greater the increase in metastability occurrence probability. We observe that if an XOR output enters a metastable state and exhibits biased bit distribution, then increasing the number of ROs would predictably lead to increased bias in the bit distribution. These results, along with Section 3.3, indicate that unless we reduce the likelihood of input signals to XOR gates entering metastable states, we cannot achieve a uniform bit distribution by increasing the number of ROs.

Now, let us consider how to reduce metastable states. Reference [20] states that when using two D-FFs for synchronization, the probability of metastability occurrence is significantly reduced. This suggests that by connecting two D-FFs after an asynchronous RO, we can effectively suppress the probability $P_{RO}\left(M\right)$ of metastable state occurrences. If we can successfully reduce the probability $P_{RO}\left(M\right)$ of metastable states, then even in the presence of biased bit distribution—as demonstrated in Section 3.3—increasing the number of ROs would approach a more uniform bit distribution.

## 4. Multi-D-FFs and Metastability

The previous section briefly reviewed the results of [17], which clarified the difference in bit distribution when connecting 0-D-FF and 1-D-FF to RO. The result shows that connecting 1-D-FF to each RO improves the bit distribution. This is thought to be because synchronizing the RO signal, which is an asynchronous noise source, with the D-FF reduces the probability that the intermediate voltage will enter the XOR gate. In this section, we investigate the difference in the bit distribution when using multi-D-FFs.

### 4.1. Synchronization Circuit

The D-FF connected after RO in the RO-based RNG has two features: sampling the analog source and synchronization with the FPGA’s onboard clock. Sampling converts an analog signal into a digital signal. An RO is an analog signal with an unstable period. For use in an FPGA, the signal is sampled by the D-FFs and transformed into a digital signal.

**Figure 6:**
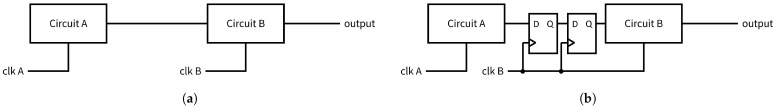
Two circuits that have respective frequencies. (**a**) An asynchronous signal is directly connected to Circuit B. (**b**) An asynchronous signal is connected to Circuit B after being synchronized by 2-D-FFs.

On the other hand, synchronization occurs when a signal that is asynchronous to the circuit’s own clock is input to the circuit. Figure 6a shows an example of the two circuits with their respective frequencies.

When signals from Circuit A are connected directly to Circuit B without proper synchronization, those signals may fail to be processed correctly within Circuit B. Many instances of improper signal processing occur when signals remain in an unstable midpoint voltage state. This condition is known as metastability, and the affected signals can significantly impact computational results. To prevent such occurrences, synchronized circuits must be implemented between asynchronous components.

The 2-D-FF circuit is normally used as a synchronization circuit [19], but Altera recommends using 3-D-FF for metastability protection [19]. Thus, when a signal synchronized with a different clock is input, a synchronization circuit constructed of two or more D-FFs must be connected, as shown in Figure 6b.

### 4.2. Proposal RO with Multi-D-FFs

In this study, we concentrated on sampling from the analog noise source in the RO-based RNG. Such an RNG uses unstable frequency as an analog noise source, which is asynchronous with the stable onboard clock. As shown in Section 3.4, if each RO is input without a synchronization circuit, these signals, which are input into an XOR gate, probably include the intermediate signal.

In the metastable state, the signal hovered in the intermediate states for some time. This phenomenon can cause computational errors and may introduce bit bias in XOR operations within RO-based RNGs. Assuming this occurrence, it would create biased bit distributions in the generated random number sequences. The work in [17] demonstrates that the RO, which is an analog noise source, must synchronize with a D-FF to improve the bit distribution of the output values from the XOR gate. Ref. [17] conducted experiments where one input of a two-input XOR gate was driven by an RO output, while the other input received a fixed bit. When the fixed-bit input was inverted, theoretically the output of the XOR gate should have reflected this change in distribution. In actuality, however, this did not occur, with the 0-D-FF showing significant percentage-point differences. Conversely, the 1-D-FF demonstrated smaller differences in these measurements. This result was attributed to bias in the XOR operation results caused by intermediate voltage inputs to the XOR gates. This shows that when the intermediate states are input to a multiple-input XOR gate, even a single one, the output is unknown, but its probability has a characteristic. Section 3.4 shows the metastable state appearance probability by the simulation. If the metastable state from the XOR gate has a bit distribution gap, it is expected to appear as a percentage-point of this experiment. Actually, previous experiments using the Nexys A7-100T board revealed that the 1 appearance has a higher percentage than the 0 appearance. These results show that the XOR calculation result in the RNG on the experimental board is likely to be 1 when inputting an intermediate potential signal, and a D-FF connection after an RO decreases the intermediate potential signal to input the XOR gate.

We considered that a decrease in the input metastability state value to an XOR gate is necessary and hypothesized that techniques such as 2-D-FF or 3-D-FF could be applied to prevent metastability, as described in [19]. Conventional methods place a 1-D-FF between the RO and XOR gates; instead, we used multi-D-FFs. The proposed method prevents the metastability from becoming an input to the XOR gate.

### 4.3. Experimental Methods

The experiment verified the effect of that the proposed method, multi-D-FFs, on the bit distribution. This verification can specifically predict the number of metastable state signals input into the XOR gate. We consider a two-input XOR gate.

One input of the two-in the XOR gate is fixed, and the other is an RO with multi-D-FFs. The 0 occurrence probability when the fixed input is 0 and the 1 occurrence probability when the fixed input is 1 should be even because the other input RO and the experimental conditions are the same. If a gap exists between these two bit distributions, it implies a bias in the output when metastable values are inputted. This experiment verifies the effect of multi-D-FFs by comparing the percentage points between the respective bit occurrence probabilities.

**Figure 7:**
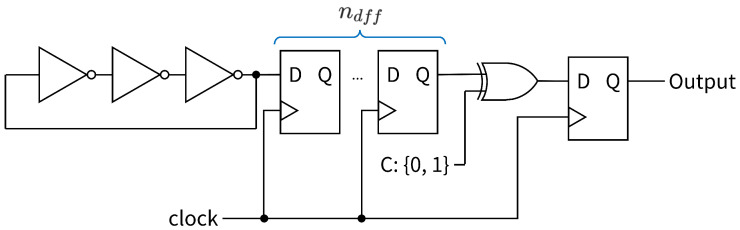
Experiment circuit: RO with multi-D-FFs.

The proposed experimental method is described as follows. The experimental circuit is shown in Figure 7. A two-input XOR gate was prepared with one input set to C∈{0,1}. The other input connects to an RO, in which three NOT gates connect the ring shape, followed by the multi-D-FFs. The number of D-FFs is denoted by $n_{dff}$, where $n_{dff}\in \left\{0,1,2,3,4\right\}$. The hardware and software environments used in the experiment are listed in Table 3.

The experimental procedure consisted of circuit implementation, sending random number data to the PC, and a random number test. The first step is the circuit implementation. The experimental circuit of Figure 7 and the UART communication circuit were designed in Verilog HDL and written to the FPGA using Vivado 2022.2. The second step is to send random number data to a PC. After running the FPGA to generate random number sequences, the sequences were sent to a PC. A 1 Kbit random number sequence was generated on the FPGA and sent to the PC via UART communication. This process was repeated to accumulate 1 Gbit of random numbers. The final step is a random number test. This study used the Markov process test [16] to measure the frequency of occurrence each bit. The Markov process test outputs the state occurrence and transition probabilies. In this case, the comparison is based on the bit occurrence probability obtained from this test. We set the value of $n_{dff}$ and conducted experiments with two different values for the constant C. For bit occurrence probability tests, we compared the difference in the probability gap between the two C patterns.

### 4.4. Results

The result is shown in Table 4. The ‘State’ shows the respective bit occurrence probabilities (%), and the ‘Gap’ represents the absolute difference in percentage points (pp) between State ‘0’ under C:1 and State ‘1’ under C:0.

Based on the results, the use of two or more D-FFs significantly reduced the gap. This indicates that increasing the number of D-FFs enables the system to converge from a metastable state to a stable bit value. Therefore, when using ROs asynchronous with the onboard clock as analog noise sources for LUT operations, it is essential to employ at least two or more D-FFs. The detailed consideration is shown in Section 6.

Through this experiment, we demonstrated that metastable states lead to bias in the XOR gate operation results and confirmed that this phenomenon can be reduced by increasing the number of D-FFs.

## 5. RO-Based RNG with Multi-D-FF Synchronization

In Section 3.2, it was confirmed that using a D-FF when feeding the RO output into an XOR gate reduces the unstable state of the XOR operation caused by the metastable states. Furthermore, it was confirmed that using two or more D-FFs further stabilized the XOR operation results. This section verifies how correctly synchronizing the RO output using D-FFs affects the randomness, specifically the bit distribution.

### 5.1. Methods

**Figure 8:**
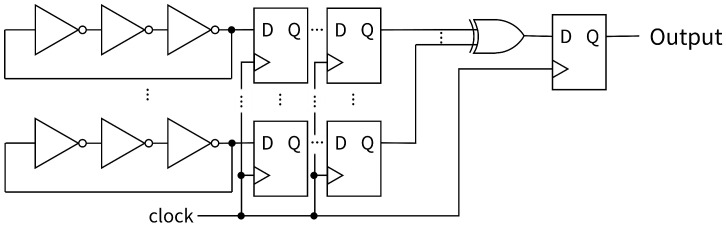
Proposed RO-based RNG with multi-D-FFs.

In this subsection, multi-D-FFs are connected between the RO and LUT, which have the same XOR gate [24] (Figure 8). We compared the randomness, especially to confirm the bit distribution, with the proposed circuit and the previous methods, which connect the 0-D-FF and the 1-D-FF.

The experimental conditions are listed in Table 3. Regarding the configuration of the RO-based RNG, the number of NOT gates for each RO was set to three, and the number of RO circuits was set to five. This experiment aimed to investigate the effects of multi-D-FFs, so it was conducted with RO.

The experimental method is explained in three steps. First, we implement the RO-based RNG module and UART communication module on the FPGA. When implementing the RO-based RNG on the FPGA, note that all RO circuit elements fit within a single SLICE. Second, the respective modules are executed on the FPGA, and the random number sequence generated by them is sent to the PC via UART communication. The sequence is generated for 1 straight Kbit in one cycle and is repeated 1,000,000 times. Thus, 1 Gbit random number data were obtained on the PC. Finally, the data were tested using NIST SP 800-22, a prominent statistical test suite for checking the randomness tests. NIST SP 800-22 is primarily a statistical randomness testing suite designed specifically for evaluating pseudorandom numbers. It establishes the ideal random number statistics as the null hypothesis and employs *p*-value assessment. When the *p*-value exceeds the significance threshold of 0.01, the null hypothesis is accepted, indicating that the sample statistics are consistent with those of the population. Additionally, $\chi^{2}$ testing confirms uniformity in *p*-values. This is achieved by applying the same test to multiple data sequences to verify whether the overall sequence exhibits any statistical biases or patterns. In this experiment, the verification target was bit distribution. Therefore, only the “Frequency test” and the “Cumulative Sums test” in NIST SP 800-22 were used as the randomness test. All NIST SP 800-22 tests were conducted using 1 Gbit of generated random data, consisting of 1000 sequences of 1 Mbit each. The significance level was set to α = 0.01, following the NIST-recommended default value.

In the verification, five types of multi-D-FFs were used between each RO and the XOR gate in the RO-based RNG. The multi-D-FFs are conventional methods, such as 0-D-FF and 1-D-FF, and the proposed methods 2-D-FFs, 3-D-FFs, and 4-D-FFs.

### 5.2. Results

The results are presented in Table 5, Table 6 and Table 7. The experiment was repeated five times, and the results of each experiment were labeled as No. 1, …, No. 5. For each experiment, the test was performed on 1000 random number sequences, and the number of sequences that passed the test out of 1000 is shown. Furthermore, the average values of the sequences that passed the test in the five experiments are presented.

**Figure 9:**
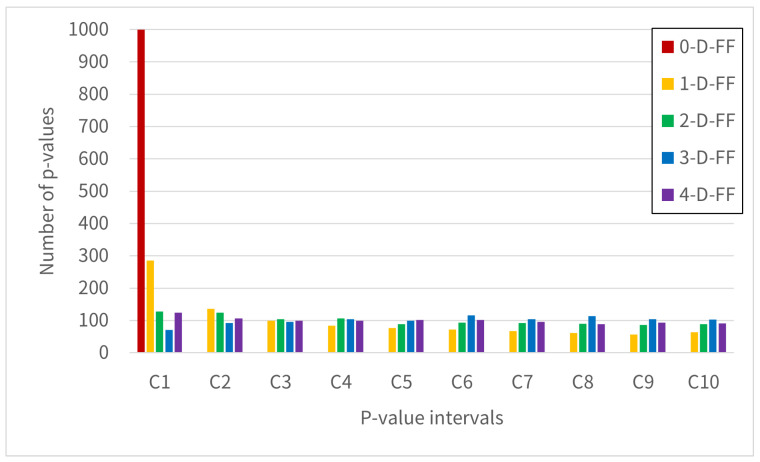
The histogram shows the 1000 *p*-values from the Frequency test, grouped by Categories C1-C10 in NIST SP 800-22.

**Figure 10:**
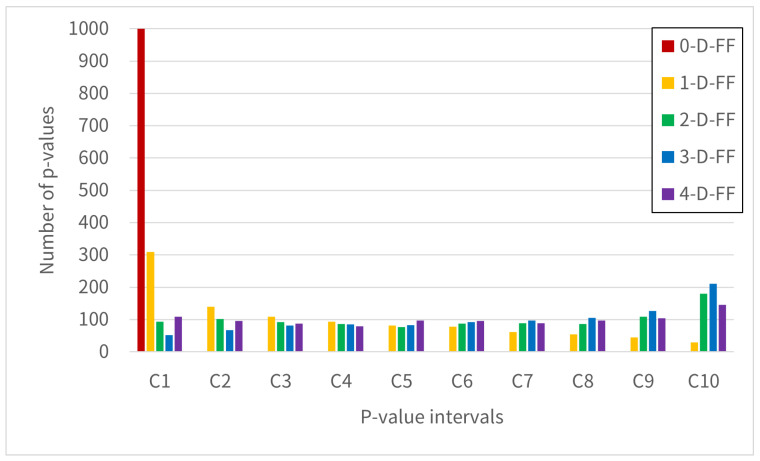
The histogram shows the 1000 *p*-values from the Cumulative Sum test (Forward), grouped by Categories C1-C10 in NIST SP 800-22.

**Figure 11:**
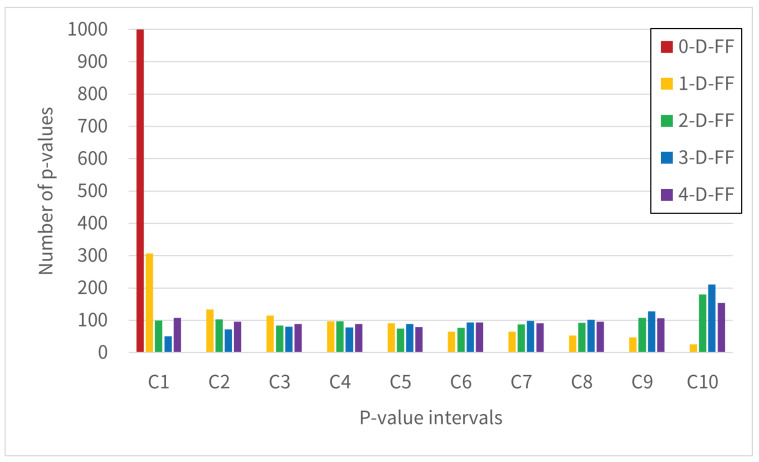
The histogram shows the 1000 *p*-values from the Cumulative Sum test (Reverse), grouped by Categories C1-C10 in NIST SP 800-22.

Figure 9, Figure 10 and Figure 11 show the distribution of test pass counts in each interval obtained by dividing the *p*-value into intervals of 0.1. The intervals shown in the graph are labeled Categories C1–C10, where C1 = [0.0, 0.1), …, C9 = [0.8, 0.9), and C10 = [0.9, 1.0].

Figure 9 shows the result of the frequency test. The result shows that for the 0-D-FF configuration, all observations appear in Category C1. For the 1-D-FF, while Category C1 appears most frequently, it is distributed broadly across Categories C2 through C10. In comparison, 2-D-FF, 3-D-FF, and 4-D-FF demonstrate a consistent uniform distribution of *p*-values across all designated categories. This section involves testing to verify whether *p*-values are uniformly distributed. The 0-D-FF result that all *p*-values appear in Category C1 stems from all tests failing. For the 1-D-FF, based on Table 2, we see an average of 924 tests passing. However, since the number of failures remains substantial, the frequency analysis reveals that although C1 appears most frequently, the distribution of *p*-values shows uniformity across the categories. The uniformly distributed results observed in 2-D-FF, 3-D-FF, and 4-D-FF indicate that these configurations satisfy the statistical characteristics of ideal random numbers.

Figure 10 and Figure 11 show the results of the cumulative sum test. Similar trends are observed to those in Figure 9. The cumulative sum test involves converting binary bits (0/1) from either the Forward or Backward orientation into (−1/+1) values for summation. A favorable test outcome indicates that the overall sequence shows minimal bias in bit distribution, while also suggesting no significant local variations in bit distribution patterns.

For these results, the RO-based RNG using 2-D-FFs improves the bit distribution over both 0-D-FF and 1-D-FF. Furthermore, 3-D-FF and 4-D-FF also yielded test results similar to those of 2-D-FF. These results demonstrate that using two or more D-FFs in the synchronization circuit of RO-based RNGs leads to improved bit distribution.

## 6. Consideration

Section 3.3 demonstrated that even with biased bit distribution inputs to an XOR gate, introducing multiple noise sources and performing XOR operations results in uniformly distributed output bits. In Section 3.4, however, we conducted simulations involving three states, including metastability, and found that the {0, 1} bit distributions in the XOR operation results did not equal 0.5. We further observed that increasing the number of noise sources elevated the probability that the output of the XOR operation would enter a metastable state. These findings demonstrate that stabilizing the input values for XOR operations leads to uniformly distributed outputs. Moreover, we confirm that when the probability of the XOR output entering a metastable state becomes dominant, it significantly increases the likelihood of biased bit distributions in the output.

These simulation results are also evident in references [17] and Section 4, where increasing the number of D-FFs resulted in smaller percentage points. This study did not actually observe metastability states or calculate the probabilities of bits converging from this state. However, we can reasonably expect that the percentage points observed in these experiments represent only a subset of cases in which the XOR gate output entered a metastable state. Thus, decreasing the percentage of cases where the output became metastable by increasing the number of synchronization circuits (D-FFs) demonstrates that asynchronous RO signals are properly synchronized with the stable on-board clock. Overall, we conclude that inserting multi-D-FFs after noise sources enables proper coupling of individual noise sources via the XOR gate, leading to an improved bit distribution as an entropy source. This result connects to the NIST SP 800-22 tests regarding bit distributions in Section 5. For 5-stage RO-based RNG implementations, we found that synchronization using either 0-D-FF or 1-D-FF resulted in biased bit distributions. However, when using two or more D-FFs, we were able to verify improved performance in both the number of passing tests for bit distribution and the uniformity test results for *p*-values.

In Section 5, we confirmed the improvement effect on the bit distribution when 2-D-FFs, 3-D-FFs, and 4-D-FFs were applied to the subsequent stage of an RO-based RNG. These results demonstrate that proper synchronization when acquiring values from the analog noise source (RO) contributes to the improvement of the bit distribution. As the technical background of the observed phenomenon, the process of acquiring data from analog noise sources requires the conversion from analog to digital information. In this conversion process, when LUTs are connected to the subsequent stage, it is considered that signals in metastable states entering the LUTs cause bias in the bit distribution. Through the application of two or more D-FFs, signal synchronization with D-FFs promotes the transition from metastable to stable states, resulting in the stabilization of the subsequent arithmetic processing.

An important point is that this approach does not modify the physical instability of the entropy source but rather aims to improve the stability of the subsequent digital signal processing section. Under the premise that unpredictable information can be properly acquired from the analog noise source, the randomness of the TRNG as a whole is improved by stabilizing subsequent processing without compromising that information.

These findings collectively confirm that employing two or more D-FFs improves the bit distribution of RO-based RNG implementations. This result can also be verified through NIST SP 800-22’s testing criteria regarding bit distributions.

## 7. Conclusions

This study mainly described the metastable state appearance at the input to an XOR gate and the bit distribution of the XOR output. We proposed inserting two or more D-FFs between each RO and the XOR gate in the RO-based TRNG and demonstrated that the bit distribution test of NIST SP 800-22 was improved. Furthermore, to evaluate the proposed method, the output random bits were simulated using the bit appearance probability when 3-state signals {0, 1, M} are input to a multiple-input XOR gate. The simulation shows an interesting result: the probability of the metastable state increases as the number of ROs increases. However, the simulation conditions need to be updated because the inputs from the respective ROs to the multiple-input XOR gate, and the respective bit variances in the stochastic process of output RO sequences, are not strictly independent.

In future work, we will use Markov state transition probabilities in the RO sequences for a more realistic simulation of the RO-based RNG. In addition, the dependence of the respective RO signals will be discussed theoretically and simulated, referring to Ref. [25]. Based on the simulation results, we will analyze the output of the RO-based RNG and compare it with the simulations. Through these studies, we will reveal the characteristics of the RO-based RNG.

## Figures and Tables

**Table 1:** Simulation environment.

Component	Specification
Host OS	Windows 11 Pro with WSL2
Guest OS	Ubuntu 24.04.2 LTS
Python Version	Python 3.12
Python Package Manager	uv 0.5.31
Python Runtime	WSL2 Ubuntu

**Table 2:** Truth Table for RO-based RNG with Metastable Signals.

RO1	RO2	XORed
0	0	0
0	1	1
1	1	0
1	0	1
1	M	M
0	M	M
M	0	M
M	1	M

**Table 3:** Experimental environment.

Category	Component	Specification
Hardware	FPGA Board	Nexys A7-100T
Software	Operating System	Windows 11 Pro
	Development Environment	Vivado 2022.2
	HDL	Verilog HDL

**Table 4:** Bit occurrence probability in RO with multi-D-FFs.

	C:0 (%)		C:1 (%)		Gap (pp)
	State ‘0’	State ‘1’	State ‘0’	State ‘1’	
0-D-FF	32.90	67.10	74.02	25.98	6.92
1-D-FF	53.84	46.16	45.99	54.01	0.17
2-D-FFs	53.88	46.12	46.14	53.86	0.02
3-D-FFs	54.38	45.62	45.69	54.31	0.07
4-D-FFs	53.48	46.52	46.53	53.47	0.01

**Table 5:** The table shows the number of passed sequences per 1000 in the frequency test in NIST SP 800-22.

	0-D-FF	1-D-FF	2-D-FFs	3-D-FFs	4-D-FFs
No.1	0	933	992	997	991
No.2	0	926	992	995	996
No.3	0	911	989	995	976
No.4	0	916	985	997	963
No.5	0	933	982	998	987
**average**	0	924	988	996	983

**Table 6:** The table shows the number of passed sequences per 1000 in the Cumulative Sum test (Forward) in NIST SP 800-22.

	0-D-FF	1-D-FF	2-D-FFs	3-D-FFs	4-D-FFs
No.1	0	927	994	999	992
No.2	0	914	992	998	990
No.3	0	908	989	998	980
No.4	0	907	989	996	969
No.5	0	921	985	998	989
**average**	0	915	990	998	984

**Table 7:** The table shows the number of passed sequences per 1000 in the Cumulative Sum test (Reverse) in NIST SP 800-22.

	0-D-FF	1-D-FF	2-D-FFs	3-D-FFs	4-D-FFs
No.1	0	933	994	998	993
No.2	0	912	994	998	995
No.3	0	905	993	996	976
No.4	0	916	989	998	966
No.5	0	922	986	998	988
**average**	0	918	991	998	984

## Data Availability

The data presented in this study are available on request from the corresponding author.
